# Biochemical Discrimination of the Down Syndrome-Related Metabolic and Oxidative/Nitrosative Stress Alterations from the Physiologic Age-Related Changes through the Targeted Metabolomic Analysis of Serum

**DOI:** 10.3390/antiox11061208

**Published:** 2022-06-20

**Authors:** Giacomo Lazzarino, Angela M. Amorini, Renata Mangione, Miriam Wissam Saab, Enrico Di Stasio, Michelino Di Rosa, Barbara Tavazzi, Giuseppe Lazzarino, Graziano Onder, Angelo Carfì

**Affiliations:** 1 UniCamillus—Saint Camillus International University of Health Sciences, Via di Sant’Alessandro 8, 00131 Rome, Italy; giacomo.lazzarino@unicamillus.org; 2 Department of Biomedical and Biotechnological Sciences, Division of Medical Biochemistry, University of Catania, Via S. Sofia 97, 95123 Catania, Italy; amorini@unict.it (A.M.A.); miriam.saab@phd.unict.it (M.W.S.); 3 Department of Basic Biotechnological Sciences, Intensive and Perioperative Clinics, Catholic University of the Sacred Heart of Rome, Largo F. Vito 1, 00168 Rome, Italy; renata.mangione@unicatt.it (R.M.); enrico.distasio@unicatt.it (E.D.S.); 4 Department of Biomedical and Biotechnological Sciences, Section of Anatomy, Histology and Movement Sciences, School of Medicine, University of Catania, Via S. Sofia 97, 95123 Catania, Italy; mdirosa@unict.it; 5 Department of Cardiovascular and Endocrine-Metabolic Diseases and Aging, Istituto Superiore di Sanità, Viale Regina Elena, 299, 00161 Rome, Italy; 6 Department of Geriatric and Orthopedic Sciences, Catholic University of Rome, Largo Francesco Vito, 1, 00168 Rome, Italy; angelo.carfi@unicatt.it; 7 Fondazione Policlinico Universitario A. Gemelli IRCCS, Largo Agostino Gemelli 8, 00168 Rome, Italy

**Keywords:** Down Syndrome, aging, targeted metabolomics, serum, mitochondrial dysfunction, energy metabolism, oxidative/nitrosative stress, HPLC

## Abstract

Down Syndrome (DS) is a neurodevelopmental disorder that is characterized by an accelerated aging process, frequently associated with the development of Alzheimer’s disease (AD). Previous studies evidenced that DS patients have various metabolic anomalies, easily measurable in their serum samples, although values that were found in DS patients were compared with those of age-matched non-DS patients, thus hampering to discriminate the physiologic age-related changes of serum metabolites from those that are truly caused by the pathologic processes associated with DS. In the present study we performed a targeted metabolomic evaluation of serum samples from DS patients without dementia of two age classes (Younger DS Patients, YDSP, aging 20–40 years; Aged DS Patients, ADSP, aging 41–60 years), comparing the results with those that were obtained in two age classes of non-DS patients (Younger non-DS Patients, YnonDSP, aging 30–60 years; Aged-nonDS Patients, AnonDSP, aging 75–90 years). Of the 36 compounds assayed, 30 had significantly different concentrations in Pooled non-DS Patients (PnonDSP), compared to Pooled DS Patients (PDSP). Age categorization revealed that 11/30 compounds were significantly different in AnonDSP, compared to YnonDSP, indicating physiologic, age-related changes of their circulating concentrations. A comparison between YDSP and ADSP showed that 19/30 metabolites had significantly different values from those found in the corresponding classes of non-DS patients, strongly suggesting pathologic, DS-associated alterations of their serum levels. Twelve compounds selectively and specifically discriminated PnonDSP from PDSP, whilst only three discriminated YDSP from ADSP. The results allowed to determine, for the first time and to the best of our knowledge, the true, age-independent alterations of metabolism that are measurable in serum and attributable only to DS. These findings may be of high relevance for better strategies (pharmacological, nutritional) aiming to specifically target the dysmetabolism and decreased antioxidant defenses that are associated with DS.

## 1. Introduction

Down Syndrome (DS) is a genetic disorder that is linked to complete or partial trisomy of chromosome 21, leading to typical physical features and intellectual disability of different grades. DS patients experience very early symptoms of premature aging processes, involving immune, respiratory, gastrointestinal, musculoskeletal, urinary, endocrine, vision and hearing systems [1], even if the real correlation between aging and DS is still controversial [2].

In addition, the premature decay of the CNS, early development of cognitive deficits [3] and AD have been documented in patients with DS [4]. The high incidence of AD that is associated with aged DS patients is due to the overexpression of the gene which is located on chromosome 21, encoding for the amyloid precursor protein (APP), the incorrect proteolytic processing of which is ultimately responsible for the overproduction of the β-amyloid peptide forming the senile plaques of AD [5,6,7]. As far as metabolism is concerned, in 1990 Lejeune et al. [8] performed studies on the biochemical aspects of DS. Theoretical considerations and clinical observations on more than 100 children with DS led the author to the conclusion that, in order to improve metabolism, it was necessary to: (1) control the dysthyroidism; (2) compensate abnormal purine derivatives; (3) equilibrate the homocysteine/methionine pathway; (4) increase folate or biopterin availability [8]. 

The genetic research located, until now, more than 300 genes on chromosome 21, including copper-zinc superoxide dismutase and cystathionine β-synthase (CBS), involved, respectively, in the increased oxidative stress [9,10] and imbalance of the methionine cycle [11,12], both characteristics of DS patients. For these reasons, several studies have been centered on evaluating the serum levels of antioxidants and biomarkers that are representative of oxidative stress [13,14], and the circulating amino acids that are involved in methylation reactions (methionine, cysteine, homocysteine, S-adenosylmethionine, S-adenosylhomocysteine, L-cystathionine) [15,16]. The importance of studying the metabolomic aspects, either to better depict the pathobiological biochemical processes that are involved in DS development, or to acquire a useful tool for targeted therapeutic protocols, has recently been underlined. In a specific study on the red blood cells of DS patients, Authors found a reprogramming in amino acid, purine metabolisms and glutathione homeostasis, suggesting specific dietary supplementations to protect the brain and procrastinate the aging of DS patients [17]. It was also underlined that the early event of the DS neuropathology might be in strict connection with iron dysregulation and the consequent mitochondrial/redox imbalance [18]. A nuclear magnetic resonance (NMR) study, carried out in the serum and urine of DS patients, pictured a large spectrum of metabolic changes involving several pathways [19] and corroborated the information of previous cross-sectional studies [11,12,16]. Alterations of the parameters that are representative of nitrosative stress, such as circulating nitrite and nitrate (as stable end-products of nitric oxide production) have also been detected in DS patients [20]. Altogether, the available information indicates that DS is biochemically characterized by the malfunctioning of the mitochondrial machinery, causing a decrease in mitochondrial oxidative metabolism, a higher glycolytic rate and consequent energy penalty. 

Parallel to this, it should be kept in mind that a large number of metabolic modifications are also present in the normal aging process [21]. It is therefore highly presumable to differentiate, even in DS, the serum metabolic changes that are related to aging from those which are connected to the pathology itself. Additionally, DS patients are characterized by accelerated aging processes, rendering somehow problematic the comparison of their serum metabolic patterns with those that are measured in age-matched non-DS subjects. Currently, there are no studies in which the results of the serum metabolites of non-DS and DS patients are evaluated by considering the age effect under physiologic and pathologic conditions, i.e., comparisons that are carried out using groups of DS and non-DS patients not matched for age. Therefore, it is yet to be clearly established whether the metabolic changes that are reported in numerous studies [9,10,11,12,13,14,15,16,17,18] are strictly characteristic of DS, rather than a part of the fluctuations that are caused by the physiologic aging process. 

In this study, using a targeted metabolomic approach, we analyzed specific classes of compounds (purines and pyrimidines, antioxidants, oxidative/nitrosative stress biomarkers, amino acids,) in the serum samples of DS patients, subsequently divided into younger (20–40 years of age) and aged (41–60 years of age). The data that were found in DS patients were compared with those that were measured in non-DS patients, subsequently divided into younger (30–60 years of age) and aged (75–90 years of age). The aim was to discriminate the potential effect of aging from those of the pathobiological processes proper of the DS on the circulating levels of the aforementioned compounds, in order to identify the metabolic pathways that are truly altered by DS, to evidence peculiar biomarkers that are exclusively attributable to DS and, therefore, useful to drive future potential DS-targeted pharmacological treatments.

## 2. Materials and Methods 

### 2.1. Study Population

The study was approved by the Ethics Committee of the Catholic University of Rome (protocol number 7437/14) and written informed consent was obtained from all patients, according to the Declaration of Helsinki.

Two groups of adult DS patients, ranging from 20 to 40 (defined as Younger DS Patients, YDSP) and from 41 to 60 (defined as Aged DS Patients, ADSP) years of age were recruited at the Day Hospital of the Area Invecchiamento, Ortopedia e Riabilitazione of the Policlinico A. Gemelli, Catholic University of Rome, Italy. In order to select the non-DS patients biologically matching the two age classes of the DS patients, two groups of non-DS patients, ranging from 30 to 60 (defined as Younger non-DS Patients, YnonDSP) and from 75 to 90 (defined as Aged non-DS Patients, AnonDSP) years of age, respectively, were also recruited at the same center, among those who were usually admitted to the Day Hospital to perform a programmed check-up. DS patients and non-DS patients’ recruitment was performed over a period of four years, between September 2015 and December 2019.

### 2.2. Inclusion and Exclusion Criteria

Fifty-six non-consecutive DS patients with an established genetic diagnosis of Down Syndrome, and 102 non-consecutive non-DS patients were included in the study. Subjects of the two groups were excluded from the study in the case of a clinical diagnosis of neurodegeneration, particularly of AD, smoking, drug assumption (including nutraceuticals) and alcohol abuse. To reduce confounding factors, all participants of each group were interviewed to assess that they had a similar dietary pattern and lifestyle (Mediterranean diet, mild-to-moderate physical activity that was compatible with the age of the subject).

### 2.3. Blood Withdrawal and Processing for Metabolite Analyses

Peripheral venous blood samples were collected, from both controls and patients, using the standard tourniquet procedure from the antecubital vein, into a single VACUETTE polypropylene tube containing a serum separator and clot activator (Greiner-Bio One GmbH, Kremsmunster, Austria). The blood withdrawals were carried out between 8.00 and 9.00 am and after at least 15 min rest before blood collection. After 30 min at room temperature, the blood withdrawals were centrifuged at 1890× *g* for 10 min and the resulting serum samples were collected and proteins removed by adding 1 mL of ice-cold far UV, HPLC-grade acetonitrile to 0.5 mL of serum, as previously described [22,23]. After vortexing for 90 s and centrifugation at 20,890× *g* for 10 min at 4 °C, supernatants were collected, supplemented with 3 mL chloroform, vigorously mixed for 120 s, and again centrifuged at 20,890× *g* for 10 min at 4 °C. The upper aqueous phase was collected and again extracted with chloroform to remove the organic solvent (acetonitrile), thus leaving protein-free aqueous serum extracts that were suitable for high performance liquid chromatographic (HPLC) analyses of metabolites. The upper aqueous phases, containing water-soluble low-molecular weight metabolites, including those under evaluation, were diluted three times with HPLC-grade water before the metabolite analyses.

### 2.4. HPLC Analysis of Purines, Pyrimidines, Antioxidants and Nitrosative Stress Biomarkers

The simultaneous isocratic HPLC separation of purines (hypoxanthine, xanthine, inosine, guanosine, uric acid); pyrimidines (uracil, β-pseudouridine, uridine); antioxidants (ascorbic acid, GSH); creatinine and nitrosative stress biomarkers (nitrite and nitrate) was performed as previously described [23,24,25]. The HPLC apparatus consisted of a Surveyor System that was connected to a highly sensitive PDA diode-array detector (Thermo Fisher Scientific Italia, Rodano, Milan, Italy), equipped with a 5-cm light-path flow cell and set up to acquire signals between 200 and 300 nm wavelengths. The data were acquired and analyzed by a PC using the ChromQuest^®^ software package that was provided by the HPLC manufacturer. Separation of the various compounds was carried out using a Hypersil 250 × 4.6 mm, 5 µm particle-size column, which was provided with its own guard column (Thermo Fisher Scientific Italia, Rodano, Milan, Italy). A separating buffer was composed by 12 mM of tetrabutylammonium hydroxide, 10 mM of KH_2_PO_4_, 0.125% methanol, pH 7.00. A flow rate of 1.2 mL/min and a column temperature of 10 °C were maintained at a constant throughout the analysis. Peak identification in deproteinized serum-sample runs was determined by matching the retention times and absorption spectra of peaks in the chromatographic runs of freshly prepared ultrapure standards. The concentration of the different compounds in the serum extracts was calculated at the wavelengths of 206 (GSH, nitrite and nitrate); 234 (creatinine); and 260 nm (purines, pyrimidines and ascorbic acid), by comparing areas of the peaks of interest with those of the chromatographic runs of standard mixtures with known concentrations.

### 2.5. HPLC Analysis of Amino Acids

The simultaneous determination of 21 primary amino group-containing compounds, including aspartate (Asp); glutamate (Glu); asparagine (Asn); serine (Ser); glutamine (Gln); histidine (His); glycine (Gly); threonine (Thr); citrulline (Cit); arginine (Arg); alanine (Ala); taurine (Tau); tyrosine (Tyr); valine (Val); methionine (Met); tryptophan (Trp); phenylalanine (Phe); isoleucine (Ile); leucine (Leu); ornithine (Orn); lysine (Lys); plus the internal standard norvaline (Norval) was performed using the pre-column derivatization of the sample with a mixture of ortophtalaldehyde (OPA) and 3-methylpropionic acid (MPA), as previously described in detail elsewhere [26]. After precolumn derivatization, 25 μL of each serum extract was loaded onto the HPLC column (Hypersil C-18, 250 × 4.6 mm, 5 μm particle size, thermostated at 21 °C) for the subsequent chromatographic separation. The separation was carried out at a flow rate of 1.2 mL/min with a step gradient that was formed by using two mobile phases with the following compositions: mobile phase A = 24 mmol/L CH_3_COONa + 24 mmol/L Na_2_HPO_4_ + 1% tetrahydrofuran + 0.1% trifluoroacetic acid, pH 6.5; mobile phase B = 40% CH_3_OH + 30% CH_3_CN + 30% H_2_O. The assignment and calculation of the OPA-derivatized amino compounds in the chromatographic runs of the serum extracts were carried out by comparing the retention times and areas of peaks with those of the peaks of the chromatographic runs of freshly-prepared ultra-pure standard mixtures with known concentrations.

### 2.6. Spectrophotometric Analysis of Serum Lactate

The spectrophotometric determination of lactate was carried out using an Agilent 89090A spectrophotometer (Agilent Technologies, Santa Clara, CA 95151, USA), following the method that was described by Artiss et al. [27]. Briefly, the reaction mixture contained 100 mM of Tris–HCl, 1.5 mM of N-ethyl-N-2-hydroxy-3-sulfopropyl-3-methylalanine, 1.7 mM of 4-aminoantipyrine, and 5 IU of horseradish peroxidase. Fifty microliters of serum were added to the mixture, let to stand for 5 min and read at 545 nm wavelength. The reaction was started with the addition of 5 IU of lactate oxidase to the cuvette (final volume = 1 mL) and it was considered ended when no change in absorbance was recorded for at least 3 min. To calculate the lactate in the serum samples, the difference in absorbance at 545 nm wavelength (ΔAbs) of each sample was interpolated with a calibration curve that was obtained by plotting ΔAbs measured in standard solutions of lactate with increasing known concentrations.

### 2.7. Statistical Analysis

The statistical analysis was performed using the GraphPad Prism program, 8.01 version. The continuous variables were expressed as mean ± SD. Since not all data displayed normal distributions, tested according to the Kolmogorov–Smirnov test, differences among the groups (PnonDSP versus PDSP) or subgroups (YnonDSP, AnonDSP, YDSP, ADSP) were determined, respectively, by the Mann–Whitney, or the Kruskal–Wallis non-parametric tests for multiple comparisons that were corrected by controlling the False Discovery Rate (FDR) using the two-stage linear step-up procedure of Benjamini, Krieger and Yekutieli. The correlation between the circulating concentrations of the different serum metabolites with the age of PnonDSP and PDSP was assessed using Spearman’s correlation coefficients. The differences were considered to be significant when *p* < 0.05 or *q* < 0.05.

## 3. Results

### 3.1. Serum Metabolites in Pooled Non-DS and Pooled DS Patients

The two groups of non-DS patients were composed of *n* = 55 subjects, mean age 44.8 ± 8.7 years, 26 females and 29 males (YnonDSP), and *n* = 47 subjects, mean age 83.2 ± 4.3 years, 22 females and 25 males (AnonDSP). Similarly, the two groups of adults DS patients were constituted of *n* = 29 subjects, mean age 29.2 ± 7.5 years, 13 females and 16 males (YDSP), and *n* = 27 subjects, mean age 51.3 ± 6.7 years, 12 females and 15 males (ADSP).

**Figure 1:**
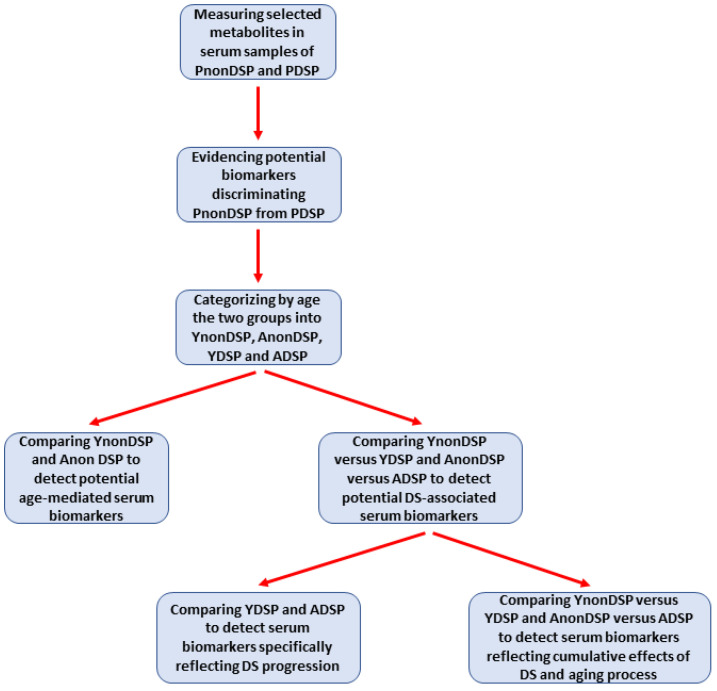
Schematic representation of the different steps (from sample analyses to statistical comparisons) allowing to achieve the main aim of the study, i.e., to discriminate DS-related from physiologic age-related changes in the circulating levels of parameters representative of oxidative/nitrosative stress and metabolic alterations, using targeted metabolomic analysis.

Figure 1 summarizes the various steps of this study, finalized to discriminate the age-related from the DS-related influence on the circulating concentrations of the parameters of interest.

The two groups of non-DS and DS patients were initially compared, regardless of the age of the subjects. Additionally, each group was also separately analyzed to determine which compounds had a correlation with the age of the subjects. Table 1 and Table 2 report the mean values of the compounds of interest in PnonDSP and PDSP, the significances of the between-groups comparison, the values of the correlation coefficients and the significances of the correlation coefficients. 

The results of this initial statistic evaluation indicate that, of the 36 compounds that were analyzed in serum, 30 of them had significantly different concentrations in PnonDSP than in PDSP (only creatinine, Gly, Ala, Val, Ile and Lys were not significantly different). In PnonDSP, changes in the serum concentrations of 11/36 compounds were positively (uracil, β-pseudouridine, Asp and Citr) or negatively (Glu, Tyr, Val, Trp, Phe, Ile and Leu) correlated with the age of the subjects, whilst in PDSP, 14/36 compounds were positively (uracil, xanthine, nitrate, nitrite + nitrate, lactate and Asp) or negatively (GSH, Glu, Met, Trp, Phe, Ile and Leu) correlated with the age of the subjects. According to this set of comparisons, it seems that PDSP have a generalized, profound imbalance of the crucial metabolic pathways involving mitochondrial functions, energy production, AA metabolism and antioxidant defenses, leading to alterations in the circulating profile of 30 biochemically relevant metabolites.

### 3.2. Serum Metabolites in Non-DS and DS Patients Categorized According to the Age of the Subjects

Since in both PnonDSP and PDSP various compounds were significantly influenced by the age of the subjects, we divided PnonDSP into YnonDSP (age ranging 30 to 60 years) and AnonDSP (age ranging 75 to 90 years), and PDSP into YDSP (age ranging 20 to 40 years) and ADSP (age ranging 41 to 60 years). The first relevant information that can be obtained from the categorization, according to the age of the subjects is that AnonDSP, compared to the concentrations that were measured in YnonDSP, had significantly different serum values of uracil, β-pseudouridine, Asp, Asn, Glu, Citr, Tau, Trp, Phe, Leu and Lys, almost fully reflecting the correlation with age that was observed in PnonDSP (Table 3 and Table 4).

The second important information (Table 3 and Table 4) is that the two sub-groups of YDSP and ADSP had different values of uracil, xanthine, uric acid, sum of oxypurines, ascorbic acid, GSH, nitrate, nitrite + nitrate, lactate, Asp, Glu, Citr, Met and Trp. Overall, these findings strongly indicate that changes in the serum levels of several metabolites (mostly amino acids) are part of the “physiological” aging process, but a conspicuous number of them (mainly purines, antioxidants and nitric oxide metabolites) represent the biochemical signature of the pathological aging process, associated with DS.

**Figure 2:**
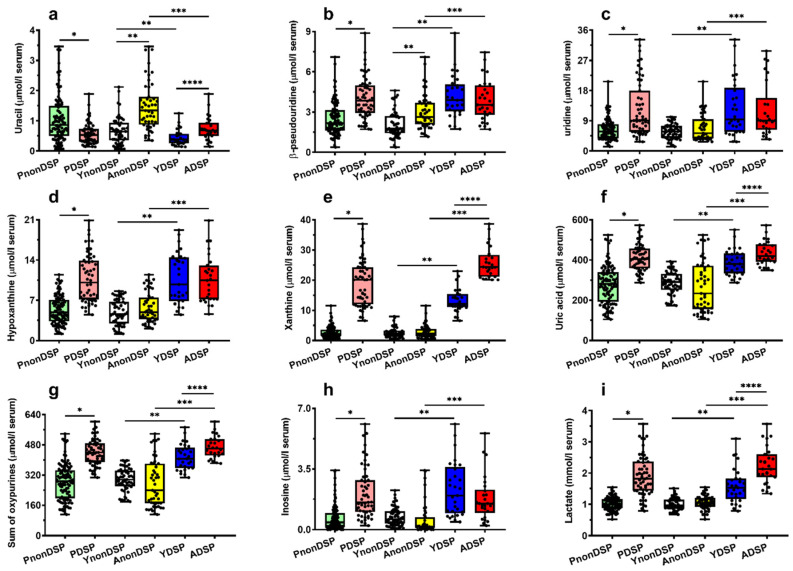
Box plots reporting all data points, 25 and 75 percentiles, minimum, maximum and median referring to the concentrations of uracil (**a**), β-pseudouridine (**b**), uridine (**c**), hypoxanthine (**d**), xanthine (**e**), uric acid (**f**), sum of oxypurines (**g**), inosine (**h**) and lactate (**i**) determined in serum samples of PnonDSP, PDSP (compared using the Mann–Whitney test), YnonDSP, AnonDSP, YDSP and ADSP (compared using the Kruskal–Wallis non-parametric tests for multiple comparisons, corrected by controlling the FDR). Sum of oxypurines = hypoxanthine + xanthine + uric acid. * *p* < 0.001; ** *q* < 0.01; *** *q* < 0.01; **** *q* < 0.01.

**Figure 3:**
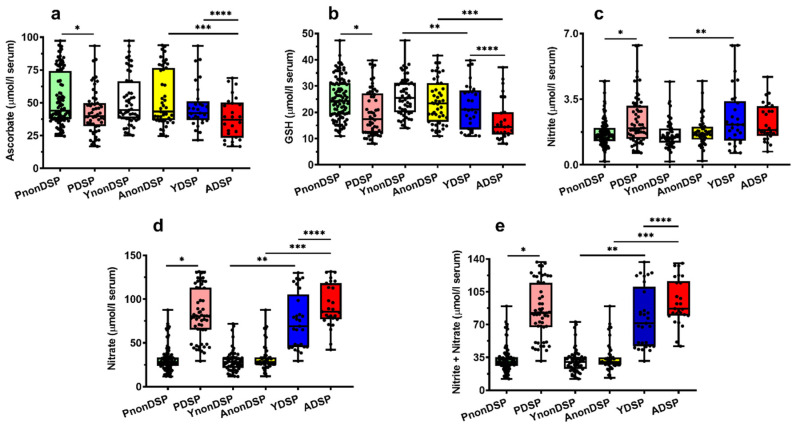
Box plots reporting all data points, 25 and 75 percentiles, minimum, maximum and median referring to the concentrations of ascorbate (**a**), GSH (**b**), nitrite (**c**), nitrate (**d**) and nitrite + nitrate (**e**) determined in serum samples of PnonDSP, PDSP (compared using the Mann–Whitney test), YnonDSP, AnonDSP, YDSP and ADSP (compared using the Kruskal–Wallis non-parametric tests for multiple comparisons, corrected by controlling the FDR). * *p* < 0.001; ** *q* < 0.01; *** *q* < 0.01; **** *q* < 0.01.

**Figure 4:**
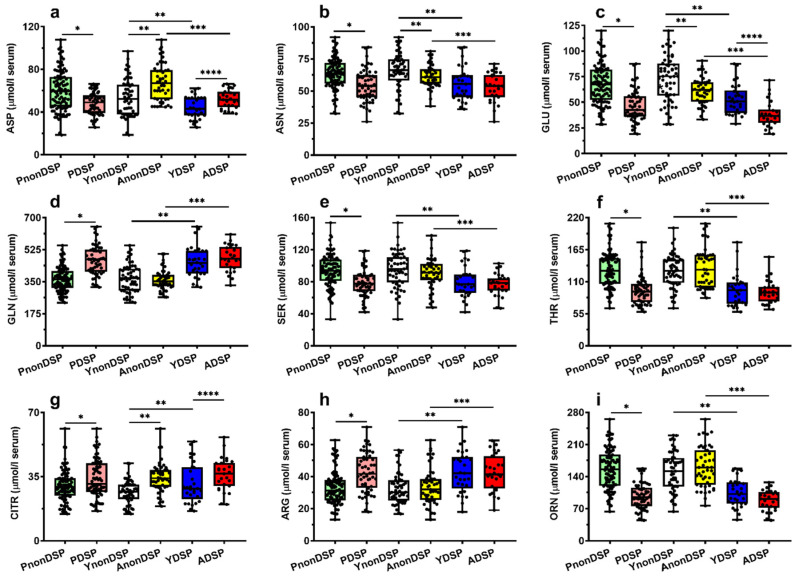
Box plots reporting all data points, 25 and 75 percentiles, minimum, maximum and median referring to the concentrations of aspartate (**a**), asparagine (**b**), glutamate (**c**), glutamine (**d**), serine (**e**), threonine (**f**), citrulline (**g**), arginine (**h**) and ornithine (**i**) determined in serum samples of PnonDSP, PDSP (compared using the Mann–Whitney test), YnonDSP, AnonDSP, YDSP and ADSP (compared using the Kruskal–Wallis non-parametric tests for multiple comparisons, corrected by controlling the FDR). * *p* < 0.001; ** *q* < 0.01; *** *q* < 0.01; **** *q* < 0.01.

**Figure 5:**
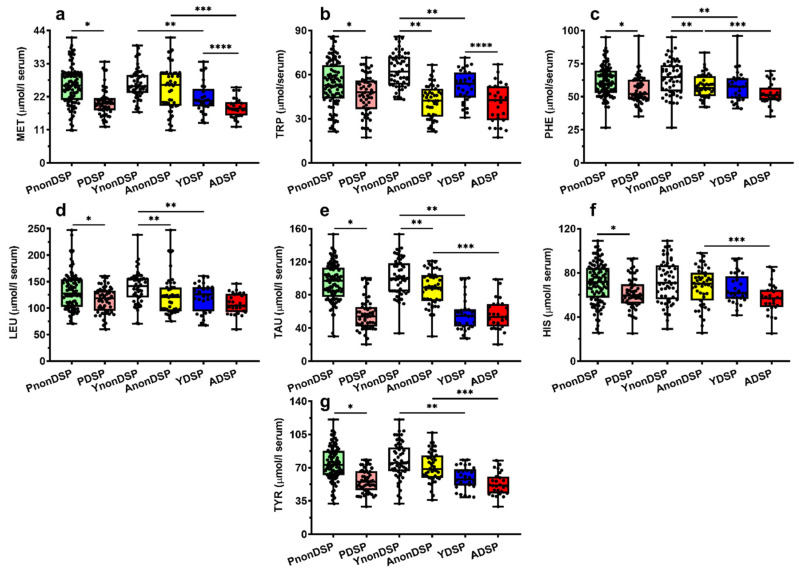
Box plots reporting all data points, 25 and 75 percentiles, minimum, maximum and median referring to the concentrations of methionine (**a**), tryptophan (**b**), phenylalanine (**c**), leucine (**d**), taurine (**e**), histidine (**f**) and tyrosine determined in serum samples of PnonDSP, PDSP (compared using the Mann–Whitney test), YnonDSP, AnonDSP, YDSP and ADSP (compared using the Kruskal–Wallis non-parametric tests for multiple comparisons, corrected by controlling the FDR). * *p* < 0.001; ** *q* < 0.01; *** *q* < 0.01; **** *q* < 0.01.

To better appreciate these observations, we reported in Figure 2, Figure 3, Figure 4 and Figure 5 the box plots showing the serum concentrations of the 30 compounds discriminating PnonDSP from PDSP, before and after the age categorization into sub-groups of the different ages (YnonDSP, AnonDSP, YDSP and ADSP). In the two subgroups of YDSP and ADSP, all the purines and pyrimidines that were quantified in the serum samples had significantly different values than those that were measured in the corresponding subgroups of YnonDSP and AnonDSP (Figure 2).

Particularly relevant appear the results for uric acid, sum of oxypurines, hypoxanthine, inosine and, above of all, the striking differences that are observed for xanthine and lactate. In the case of circulating antioxidants and nitrosative stress biomarkers, no age-mediated differences were observed between YnonDSP and AnonDSP. Whilst ascorbate differed only between AnonDSP and ADSP and nitrite between YnonDSP and YDSP, the serum concentrations of GSH, nitrate and nitrite + nitrate in YnonDSP and AnonDSP were significantly different, respectively, from those that were recorded in YDSP and ADSP (Figure 3). Therefore, the decreased serum levels of both water-soluble serum antioxidants (ascorbate and GSH) and increased nitrosative stress biomarkers indicate diminished circulating antioxidant defenses and increased nitric oxide production in the age-divided sub-groups of DS patients.

Different findings were seen in the case of circulating amino acids. The serum concentrations in YnonDSP and AnonDSP of Citr and of the four biochemically interconnected amino acids Asp, Glu and Asn (Figure 3), as well as those of Trp, Phe, Leu, and of the amino-sulphur containing compound Tau, showed significant differences that were caused by the age of the subjects (Figure 4 and Figure 5).

Age-mediated differences between YDSP and ADSP were found only in the case of Asp, Glu, Citr, Met and Trp. The subsequent comparisons allowed to show that Asp, Glu, Asn, Gln, Ser, Thr, Arg, Orn, Met, Phe, Tau and Tyr had significantly different circulating concentrations in YnonDSP, compared to YDSP, and in AnonDSP, compared to ADSP; Citr, Trp and Leu were statistically different only in YnonDSP, compared to YDSP, and His was significantly different only in AnonDSP, compared to ADSP.

### 3.3. Serum Metabolites Specific of DS

According to the lack of age-mediated influence in non-DS subjects (no differences in any of the following parameters between YnonDSP and AnonDSP), it is possible to affirm that the significant alterations in the serum concentrations of uridine, hypoxanthine, xanthine, uric acid, sum of oxypurines, inosine, lactate, ascorbic acid, GSH, nitrate, nitrite + nitrate, Gln, Ser, Thr, Arg, Orn, Met, His and Tyr that are found in PDSP, YDSP and ADSP (Table 1, Table 2, Table 3 and Table 4 and Figure 1, Figure 2, Figure 3 and Figure 4 ) are specifically connected to the dysmetabolisms proper of DS, rather than to metabolic alterations accompanying the physiologic aging process. Furthermore, some of these metabolites (xanthine, uric acid, sum of oxypurines, lactate, ascorbic acid, GSH, nitrate, nitrite + nitrate and Met) had significantly different concentrations when comparing the values that were found in the serum of YDSP with those that were measured in ADSP. It is however important to note that even the compounds that were affected by the physiologic aging process (Asp, Asn, Glu, Citr, Trp, Phe, Leu and Tau) suffered the influence of the pathological, DS-associated aging, i.e., serum levels of the aforementioned metabolites undergo changes, cumulating a “pathological” component that is linked to DS with the “physiological” effects of aging.

**Figure 6:**
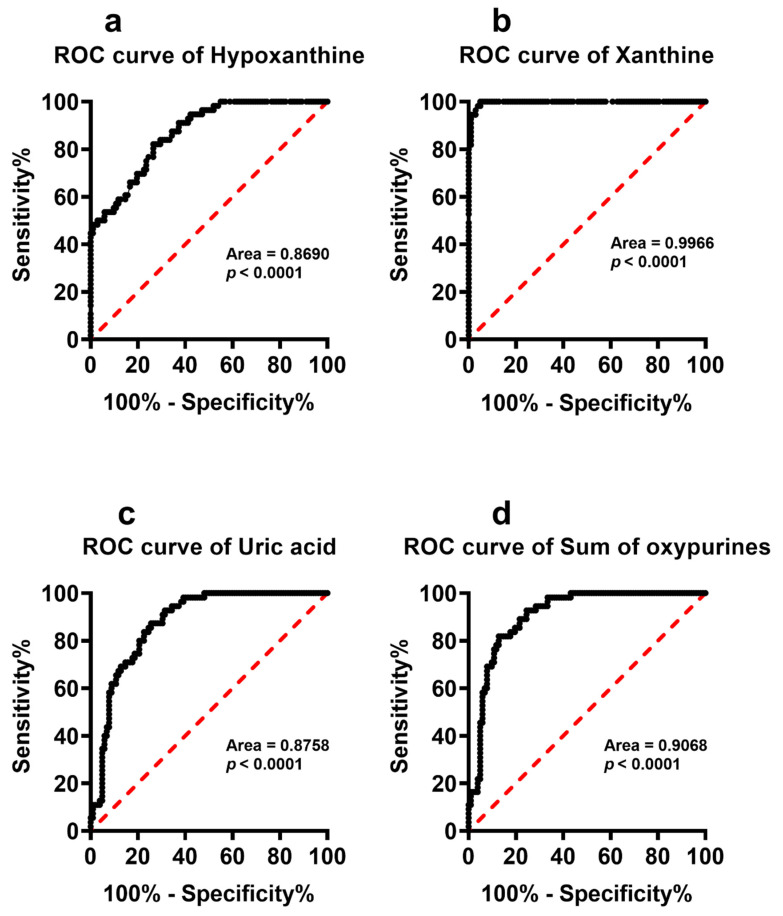
Receiver Operating Characteristic (ROC) curves calculated using the circulating levels of hypoxanthine (**a**), xanthine (**b**), uric acid (**c**) and sum of oxypurines (**d**) determined in serum samples of PnonDSP and PDSP.

**Figure 7:**
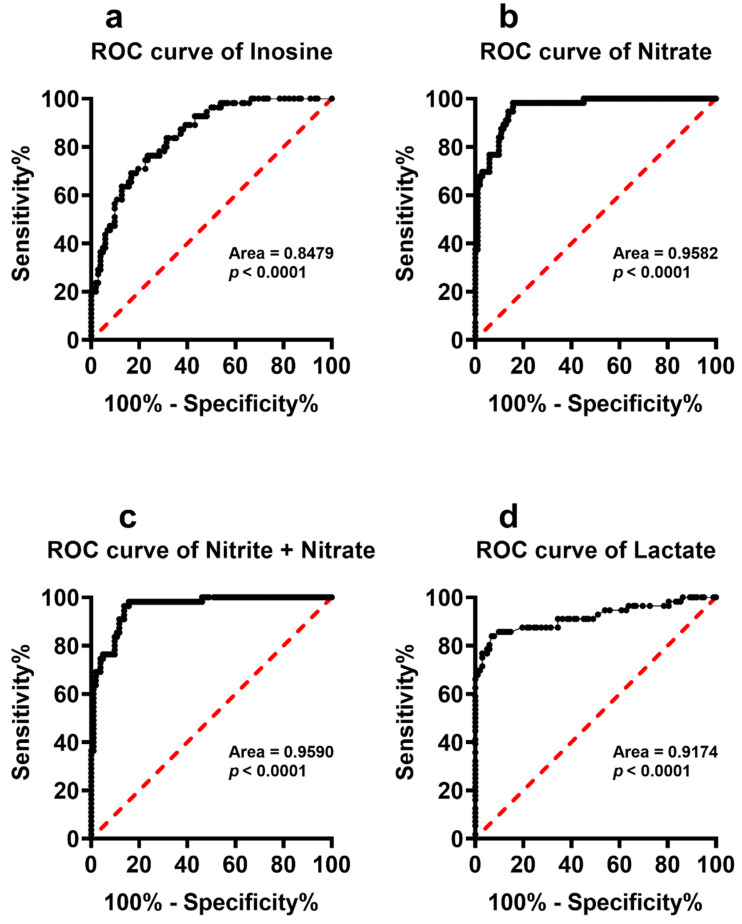
Receiver Operating Characteristic (ROC) curves calculated using the circulating levels of inosine (**a**), nitrate (**b**), nitrite + nitrate (**c**) and lactate (**d**) determined in serum samples of PnonDSP and PDSP.

**Figure 8:**
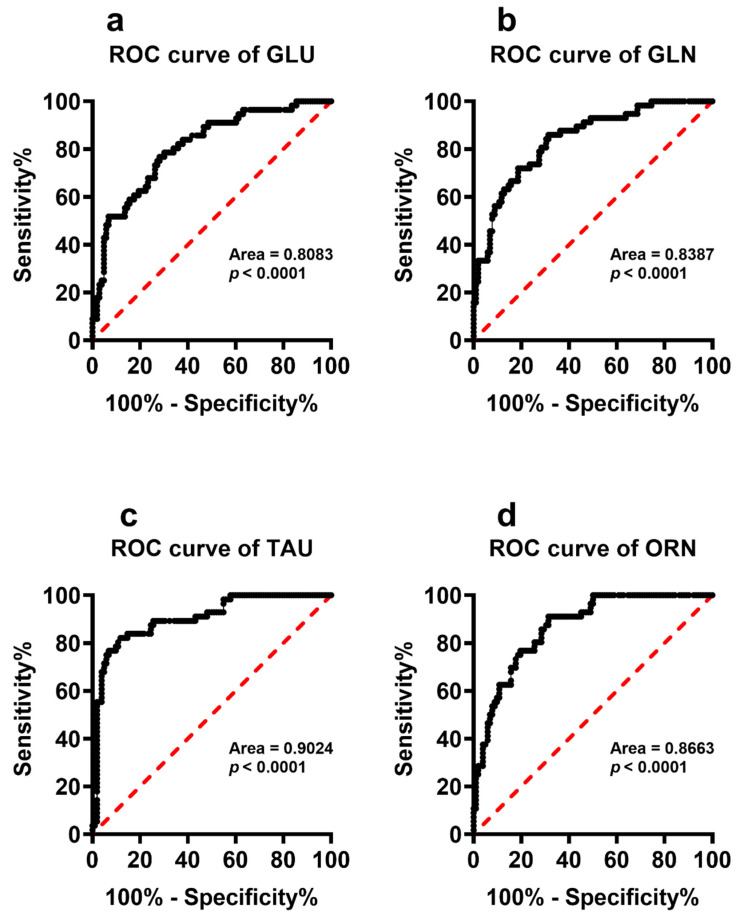
Receiver Operating Characteristic (ROC) curves calculated using the circulating levels of glutamate (**a**), glutamine (**b**), taurine (**c**) and ornithine (**d**) determined in serum samples of PnonDSP and PDSP.

To better evaluate whether any of the serum metabolites with significantly different serum concentrations in PnonDSP and PDSP may discriminate the subjects into two distinct groups we calculated the ROC curves that are illustrated in Figure 6, Figure 7 and Figure 8.

It is particularly evident that most of the compounds that were specifically affected by DS (hypoxanthine, xanthine, uric acid, sum of oxypurines, inosine, nitrate, nitrite + nitrate, lactate, Glu, Gln, Tau and Orn) were characterized by very high sensitivity and specificity and cluster PnonDSP and PDSP in two distinct groups (Figure 6, Figure 7 and Figure 8).

It is worth mentioning that even Glu and Tau, notwithstanding that both were different in YnonDSP, compared to AnonDSP, can specifically and selectively differentiate PnonDSP from PDSP (Figure 8).

**Figure 9:**
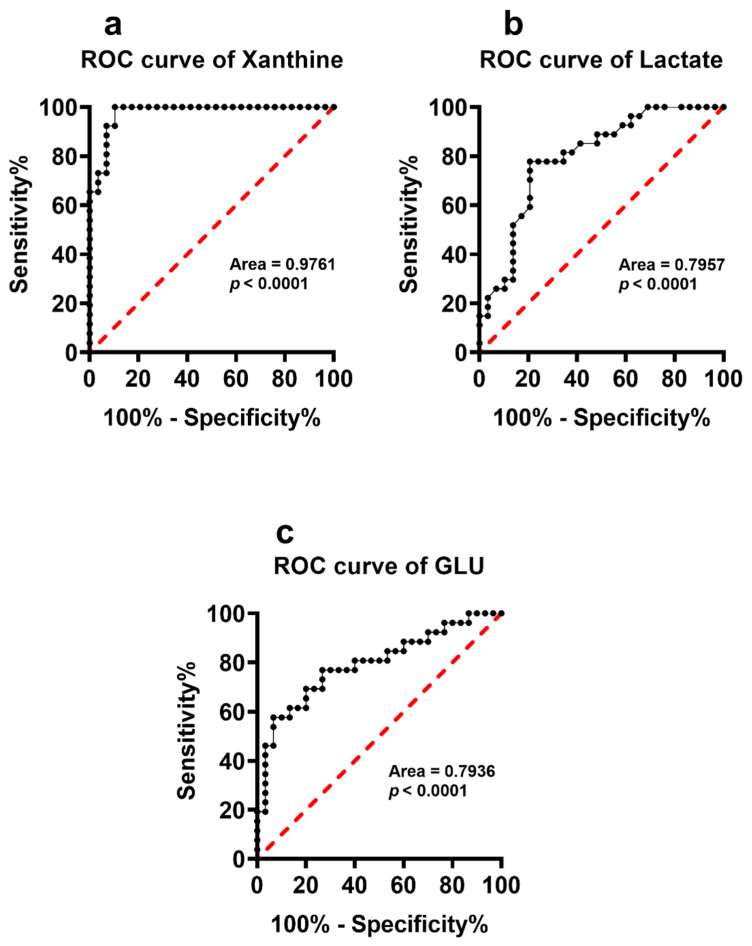
Receiver Operating Characteristic (ROC) curves calculated using the circulating levels of xanthine (**a**), lactate (**b**) and glutamate (**c**) determined in serum samples of YDSP and ADSP.

When performing the calculations of the ROC curves to determine whether any of the measured metabolites were capable of discriminating YDSP from ADSP we found that only xanthine, lactate and Glu had good indexes of both selectivity and specificity (Figure 9). 

## 4. Discussion

By using a non-DS group of patients, composed of two subgroups of young and aged non-DS subjects, the results of this study demonstrate that: (i) compared to PnonDSP, PDSP have a profound imbalance of metabolism altering the circulating levels of numerous compounds that are related to the metabolism of purines, pyrimidines, antioxidants, nitric oxide, glucose and amino acids; (ii) non-DS patients show significant changes in the serum concentrations of various metabolites, mostly amino acids, when divided into the age-related sub-groups of YnonDSP and AnonDSP; (iii) the comparisons of the age-categorized subgroups of YDSP and ADSP, with the corresponding sub-groups of YnonDSP and AnonDSP, evidence that a conspicuous number of the serum metabolite alterations that are found in DS patients are not imputable to the pathology itself, but rather to the physiologic aging process of human beings; (iv) a cumulative effect of the “pathological” DS-associated alterations with the “physiological” age-mediated modifications exacerbates changes in the serum levels of several metabolites (mostly amino acids) in DS patients. 

In a recent extensive review, Pecze et al. [28] examined the results of clinical studies in which, among others, metabolic patterns in different biofluids, including serum were determined. Serum/plasma uric acid [29,30,31,32,33,34,35,36,37,38], lactate [19,38,39,40] and different amino acids [19,38,39,40] were found to be significantly different in DS patients, compared to the values that were measured in controls, similarly to what we obtained when comparing the data of our groups of PnonDSP and PDSP. However, the aforementioned studies did not take into account the possibility of an age-mediated influence on the circulating levels of the metabolites that were assayed since, as control groups, age- and sex-matched non-DS subjects were always used.

For the first time and to the best of our knowledge, the results that were reported in the present targeted metabolomic study allowed to discriminate physiologic age-related from pathologic DS-related alterations on 30/36 serum metabolites, as well as to evidence how aging in DS patients affects the circulating levels of some of these compounds, in a different manner from what occurs during the aging of non-DS patients. 

In the initial comparison of our data, all the compounds that were related to purine and pyrimidine metabolism (hypoxanthine, xanthine, uric acid, inosine, uracil, β-pseudouridine, uridine); antioxidants (ascorbic acid and GSH); nitric oxide metabolism (nitrite and nitrate); and energy metabolism (lactate) showed significantly different concentrations in the serum samples of PnonDSP, compared to those that were measured in PDSP (Table 1). The subsequent categorization of PnonDSP evidenced that only uracil and β-pseudouridine had different values in YnonDSP, compared to AnonDSP (Table 3). Therefore, according to this finding, the serum changes of hypoxanthine, xanthine, uric acid, sum of oxypurines, inosine, ascorbic acid, GSH, nitrate, nitrite + nitrate and lactate are associated with DS, truly reflecting a pathological dysmetabolism rather than a physiologic, age-related modification of metabolism. 

Since the initial findings of Lejeune et al. [8], the imbalance of purine metabolism in DS patients has been found in various studies [29,30,31,32,33,34,35,36,37,38] and is considered to be a typical biochemical serum/plasma signature of this pathological state. Our data, besides confirming previous results [8,29,30,31,32,33,34,35,36,37,38], add a relevant new piece of information, since we found that alterations of serum xanthine, uric acid and sum of oxypurines were significantly higher in the sub-group of ADSP than in that of YDSP, indicating purine dysmetabolism worsening with the increasing age of DS patients. Interestingly, the sub-group of ADSP also had significantly higher values of nitrate, nitrite + nitrate and lactate, and lower concentrations of ascorbic acid and GSH than the corresponding values that were determined in the serum of YDSP. The concomitant increase in circulating purines and lactate strongly suggests that DS patients suffer from mitochondrial dysfunction, leading to alterations of oxidative metabolism, causing an imbalance between ATP production and consumption and, ultimately, energy penalty. In turn, these phenomena should be responsible either for the increased rate of the catabolic pathway of purine degradation [41], causing higher levels in their serum concentrations [42], or for the increased rate of glycolysis to compensate the decrease in mitochondrially produced ATP [43,44], causing higher levels in serum lactate [45,46]. The relevant finding is that, since these biochemical dysfunctions worsened by the increasing age of DS patients, they may significantly contribute to the accelerated aging process, with the deterioration of mental status that is characteristic of DS [47,48]. 

It is worth recalling that altered circulating concentrations of purines, lactate, antioxidants and nitric oxide metabolites have been recorded in patients that were affected by chronic neurodegenerations, including multiple sclerosis [42,49], AD [50,51,52,53], Parkinson’s disease [54,55,56,57], as well as in stroke [58,59,60,61], and myocardial ischemia and reperfusion [62,63,64,65,66]. The common features of these pathological conditions are mitochondrial dysfunction with energy metabolism imbalance and the presence of sustained oxidative/nitrosative stress. This implies that changes in the serum levels of purines, lactate, antioxidants and nitric oxide metabolites (nitrate + nitrate) reflect the alterations of numerous biochemical functions in DS patients, potentially contributing to their accelerated aging processes [67] and to the associated decline in their neurocognitive functions [68]. It is significant to observe that the ROC curves, calculated for purines (hypoxanthine, xanthine, uric acid, sum of oxypurines and inosine), lactate, and nitric oxide metabolites (nitrate and nitrate + nitrate) have high specificity and sensitivity to discriminate PnonDSP from PDSP, whilst only the ROC curve of xanthine allows the discrimination of YDSP from ADSP. These results strongly corroborate previous findings showing impaired mitochondrial functions [69] with energy penalty [70,71,72], not only in brain [73], but also in myocardial cells of DSP [74].

When considering the initial comparison of the circulating concentrations of amino acid levels between PnonDSP and PDSP, only 5/21 of them (Gly, Ala, Val, Ile and Lys) were not significantly different. However, the further comparisons of the age-divided sub-groups of non-DS patients into YnonDSP and AnonDSP revealed that the serum levels of Asp, Asn, Glu, Citr, Trp, Phe, Leu and Tau changed, according to the age of the two sub-groups, revealing that only the serum changes of Gln, Ser, Thr, Arg, Orn, Met, His and Tyr are directly connected to DS. Previous studies reported anomalies of various amino acids in the serum samples of DS patients, compared to those that were measured in non-DS patients [19,38,39,40]. Again, in none of these studies was the accelerated aging process characterizing DS patients considered [47,48,67], so that the comparisons were performed between groups of non-DS and DS patients that were matched for sex and, most importantly, age. 

Our results therefore demonstrate that DS patients suffer from a restricted number of anomalies that are related to amino acid metabolism, affecting important biochemical functions which are connected to neurotransmission (Gln, Ser, Thr, Tyr), energy metabolism (Ser and Thr), nitric oxide production (Arg and Orn) and methylation reactions (Met). It is however worth underlining that even the modifications occurring in AnonDSP (Asp, Asn, Glu, Citr, Trp, Phe, Leu and Tau), compared to those of YnonDSP are amplified by DS, thus again indicating a cumulative effect of the age-related “physiologic” changes with the DS-related “pathologic” changes of amino acid metabolism. Among these compounds, Glu is the only amino acid for which significant ROC curves, discriminating both PnonDSP from PDSP and YDSP from ADSP with good specificity and sensitivity were obtained. The crucial role of Glu homeostasis in DS has previously been demonstrated in a study in which we found remarkably lower Glu and higher Gln values in the amniotic fluid samples of women carrying DS fetuses, compared to those that were measured in women carrying non-DS fetuses [75].

## 5. Conclusions

**Figure 10:**
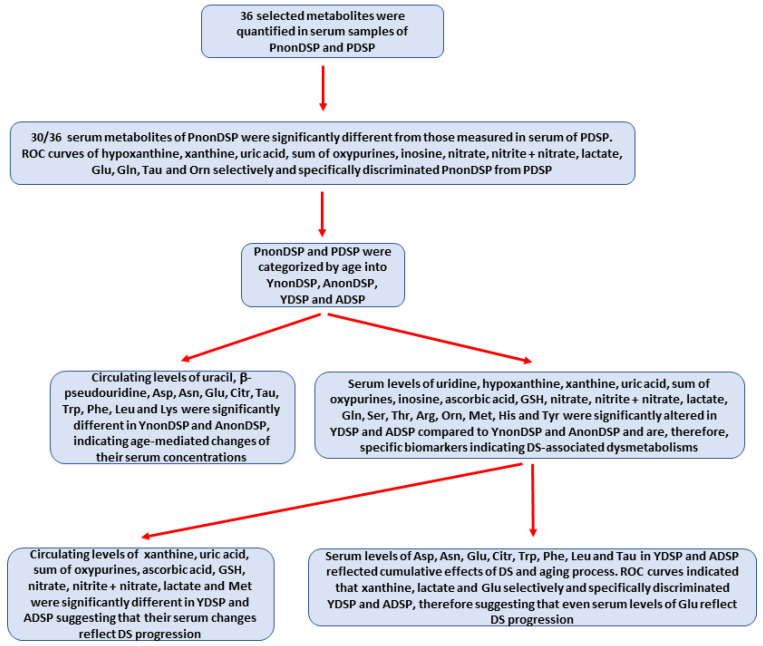
Schematic representation of the most salient findings and observations unveiled by the study.

In conclusion, the findings that were unveiled by this study, the most salient of which are schematically summarized in Figure 10, allowed to discriminate the physiologic, age-related from the pathologic, DS-related changes of metabolism through a targeted metabolomic approach analyzing selected low-molecular weight compounds in serum samples. 

These data strongly demonstrate that the results of the analysis of selected circulating metabolites, useful to characterize and monitor DS patients, should not be compared with aged-matched non-DS subjects, but rather with groups of non-DS patients of increasing age. The reason for this choice is to render the two groups to compare similar from a biological point of view, stating the accelerated aging process that is associated with DS. Additionally, the present findings indicate that, among the metabolites that were assayed in our serum samples, only purines, lactate, water-soluble antioxidants, nitric oxide metabolites and a few specific amino acids are useful either to distinguish DS from non-DS patients of different age, or to characterize the pathologic aging effect occurring in DS patients. Lastly, the biochemical pattern of specific serum metabolite alterations clearly evidenced profound metabolic dysfunctions, mainly linked to impaired mitochondrial activity (causing both energy penalty and an increase in oxidative/nitrosative stress) and the homeostasis of selected amino acids (potentially affecting neurotransmission, energy metabolism and methylation reactions). Future studies are needed to verify whether the aforementioned biochemical functions/pathways might positively be affected by selected diets/treatments in DS.

## Figures and Tables

**Table 1:** Values of the circulating concentrations (means ± SD) of representative compounds of pyrimidine (uracil, β-pseudouridine, uridine) and purine (hypoxanthine, xanthine, uric acid, sum of oxypurines, inosine) metabolism; of antioxidant defenses (ascorbic acid, GSH) and of nitric oxide metabolism (nitrite, nitrate, nitrite + nitrate) in PnonDSP (spanning 30 to 90 years of age) and PDSP (spanning 20 to 60 years of age). Significances of the comparisons of the values of each compound measured in the two groups are indicated. Correlation coefficients of each compound with the age of the subjects, as well as the resulting significances are reported.

Compound	Concentrations in Serum of PnonDSP (*n* = 102) μmol/L Serum	Concentrations in Serum of PDSP (*n* = 56) μmol/L Serum	Significantly Different from PnonDSP *q*-Values	Correlation with the Age of the Subject, Values of the Spearman’s Correlation Coefficient r of PnonDSP	*p*-Values of r of PnonDSP	Correlation with the Age of the Subject, Values of the Spearman’s Correlation Coefficient r of PDSP	*p*-Values of r of PDSP
Uracil	1.05 ± 0.76	0.59 ± 0.36	<0.0001	0.456	<0.0001	0.378	<0.005
β-pseudouridine	2.50± 1.26	4.03 ± 1.54	<0.0001	0.359	<0.0002	0.011	N.S.
Uridine	6.39 ± 3.23	12.64 ± 8.20	<0.0001	0.063	N.S.	0.104	N.S.
Hypoxanthine	5.40 ± 2.40	10.73 ± 4.22	<0.0001	0.076	N.S.	0.125	N.S.
Xanthine	2.86 ± 1.89	19.18 ± 7.76	<0.0001	−0.028	N.S.	0.299	<0.05
Uric acid	280.25 ± 101.71	410.40 ± 67.72	<0.0001	−0.086	N.S.	0.248	N.S.
Sum of oxypurines	290.96 ± 104.67	439.65 ± 70.63	<0.0001	−0.059	N.S.	0.243	N.S.
Inosine	0.77 ± 0.66	2.15 ± 1.55	<0.0001	−0.055	N.S.	0.044	N.S.
Ascorbic acid	52.50 ± 20.33	42.72 ± 17.02	<0.002	0.010	N.S.	−0.175	N.S.
GSH	25.10 ± 7.83	19.67 ± 8.75	<0.001	−0.146	N.S.	−0.392	<0.005
Nitrite	1.72 ± 0.84	2.38 ± 1.32	<0.002	0.049	N.S.	0.031	N.S.
Nitrate	31.37 ± 14.09	83.30 ± 29.70	<0.0001	0.044	N.S.	0.392	<0.005
Nitrite + Nitrate	33.09 ± 14.23	85.68 ± 29.70	<0.0001	0.037	N.S.	0.392	<0.005
Lactate	1.05 ± 0.23	1.91 ± 0.65	<0.0001	0.192	N.S.	0.529	<0.0001
Creatinine	69.89 ± 26.81	75.91 ± 18.41	N.S.	0.101	N.S.	0.054	N.S.

Sum of oxypurines = hypoxanthine + xanthine + uric acid; GSH = reduced glutathione; PnonDSP = pooled non-DS Patients; PDSP = pooled DS Patients; N.S. = not significant.

**Table 2:** Values of the circulating amino acids concentrations (means ± SD) in PnonDSP (spanning 30 to 90 years of age) and PDSP (spanning 20 to 60 years of age). Significances of the comparisons of the values of each compound measured in the two groups are indicated. Correlation coefficients of each compound with the age of the subjects, as well as the resulting significances are reported.

Compound	Concentrations in Serum of PnonDSP (*n* = 102) μmol/L serum	Concentrations in Serum of PDSP (*n* = 56) μmol/L serum	Significantly Different from PnonDSP *q*-values	Correlation with the Age of the Subject, Values of the Spearman’s Correlation Coefficient r of PnonDSP	*p*-Values of r of PnonDSP	Correlation with the Age of the Subject, Values of the Spearman’s Correlation Coefficient r of PDSP	*p*-Values of r of PDSP
ASP	60.15 ± 18.88	47.85 ± 10.38	<0.0001	0.316	<0.002	0.318	<0.02
GLU	67.45± 19.22	46.08 ± 15.55	<0.0001	−0.275	<0.002	−0.447	<0.001
ASN	63.65 ± 11.43	54.19 ± 12.25	<0.0001	−0.118	N.S.	−0.156	N.S.
SER	93.03 ± 20.74	78.75 ± 17.42	<0.0001	−0.030	N.S.	−0.088	N.S.
GLN	363.09 ± 68.14	467.40 ± 78.58	<0.0001	−0.203	N.S.	0.229	N.S.
HIS	73.68± 14.89	62.11 ± 14.04	<0.0001	−0.077	N.S.	−0.257	N.S.
GLY	220.41 ± 79.08	212.07 ± 49.26	N.S.	−0.161	N.S.	0.181	N.S.
THR	131.08 ± 30.53	95.68 ± 25.24	<0.0001	−0.082	N.S.	−0.121	N.S.
CITR	29.86 ± 8.52	33.89 ± 10.44	<0.02	0.414	<0.0001	0.227	N.S.
ARG	33.11 ± 10.34	42.48 ± 12.01	<0.0001	0.102	N.S.	−0.076	N.S.
ALA	371.96 ± 96.49	382.45 ± 106.78	N.S.	−0.136	N.S.	0.221	N.S.
TAU	95.23 ± 22.49	56.50 ± 19.03	<0.0001	−0.178	N.S.	−0.033	N.S.
TYR	74.30± 17.82	56.29 ± 12.33	<0.01	−0.203	<0.05	−0.260	N.S.
VAL	263.10 ± 59.97	248.92 ± 46.79	N.S.	−0.474	<0.0001	−0.213	N.S.
MET	25.58 ± 6.40	20.10 ± 4.60	<0.0001	−0.164	N.S.	−0.277	<0.05
TRP	53.45± 15.68	46.71± 13.26	<0.0001	−0.636	<0.0001	−0.517	<0.0001
PHE	61.44± 11.62	54.99± 10.32	<0.0001	−0.244	<0.02	−0.289	<0.05
ILE	69.63 ± 20.66	63.84 ± 13.99	N.S.	−0.266	<0.01	−0.299	<0.03
LEU	132.76 ± 34.49	115.73 ± 24.23	<0.02	−0.396	<0.0001	−0.283	<0.05
ORN	156.00 ±43.57	96.76 ±28.38	<0.0001	0.077	N.S.	−0.163	N.S.
LYS	204.68 ± 47.71	209.80 ± 45.10	N.S.	−0.072	N.S.	−0.096	N.S.

ASP = aspartate, GLU = glutamate, ASN = asparagine, SER = serine, GLN = glutamine, HIS = histidine, GLY = glycine, THR = threonine, CIT = citrulline, ARG = arginine, ALA = alanine, TAU = taurine, TYR = tyrosine, VAL = valine, MET = methionine, TRP = tryptophan, PHE = phenylalanine, ILE = isoleucine, LEU = leucine, ORN = ornithine, LYS = lysine; PnonDSP = pooled non-DS Patients; PDSP = pooled DS Patients; N.S. = not significant.

**Table 3:** Values of the circulating concentrations (means ± SD) of representative compounds of pyrimidine (uracil, β-pseudouridine, uridine) and purine (hypoxanthine, xanthine, uric acid, sum of oxypurines, inosine) metabolism; of antioxidant defenses (ascorbic acid, GSH); and of nitric oxide metabolism (nitrite, nitrate, nitrite + nitrate) in YnonDSP (spanning 30 to 60 years of age), AnonDSP (spanning 75 to 90 years of age), YDSP (spanning 20 to 40 years of age) and ADSP (spanning 41 to 60 years of age). Significances of the comparisons between YnonDSP and AnonDSP, and YDSP and ADSP of the values of each compound are indicated.

Compound	Concentrations in Serum of YnonDSP (*n* = 55) μmol/L Serum	Concentrations in Serum of AnonDSP (*n* = 47) μmol/L serum	Significantly Different from YnonDSP *q*-Values	Concentrations in Seraum of YDSP (*n* = 29) μmol/L Serum	Concentrations in Serum of ADSP (*n* = 27) μmol/L Serum	Significantly Different from YDSP *q*-Values
Uracil	0.69 ± 0.54	3.01 ± 1.34	<0.0001	0.44 ± 0.26	0.75 ± 0.39	<0.002
β-pseudouridine	2.13 ± 1.04	1.53 ± 1.07	<0.005	4.17 ± 1.58	3.89 ± 1.53	N.S.
Uridine	5.71 ± 2.38	7.07 ± 4.01	N.S.	12.78 ± 8.38	12.48 ± 8.16	N.S.
Hypoxanthine	4.95 ± 2.09	5.74 ± 2.66	N.S.	10.81 ± 4.39	10.64 ± 4.12	N.S.
Xanthine	2.74 ± 1.57	3.00 ±2.21	N.S.	13.24 ± 3.86	25.80 ± 5.18	<0.02
Uric acid	285.02 ± 55.91	272.40 ± 136.47	N.S.	391.26 ± 69.54	430.95 ± 60.38	<0.03
Sum of oxypurines	292.20 ± 56.55	286.87 ± 141.34	N.S.	417.45 ± 72.74	463.49 ± 60.95	<0.02
Inosine	0.76 ± 0.51	0.79 ± 0.81	N.S.	2.32 ± 1.57	1.97 ± 1.53	N.S.
Ascorbic acid	51.08 ± 18.67	53.22 ± 21.37	N.S.	47.27 ± 17.19	37.83 ± 15.71	<0.05
GSH	26.13 ± 6.96	23.97 ± 8.40	N.S.	22.26 ± 8.75	16.88 ± 8.00	<0.02
Nitrite	1.64 ± 0.70	1.82 ± 0.93	N.S.	2.49 ± 1.58	2.27 ± 0.99	N.S.
Nitrate	30.35 ± 12.72	33.06 ± 15.22	N.S.	74.70 ± 31.57	92.53 ± 24.93	<0.001
Nitrite + Nitrate	31.99 ± 12.78	34.88 ± 15.42	N.S.	77.19 ± 31.87	94.80 ± 24.59	<0.01
Lactate	1.13 ± 0.30	1.05 ± 0.22	N.S.	1.62 ± 0.58	2.16 ± 0.62	<0.001
Creatinine	63.47 ± 16.20	75.88 ± 34.95	<0.03	73.15 ± 20.46	78.86 ± 15.77	N.S.

Sum of oxypurines = hypoxanthine + xanthine + uric acid; GSH = reduced glutathione; YnonDSP = Young non-DS Patients; AnonDSP = Aged non-DS Patients; YDSP = Young DS Patients; ADSP = Aged DS Patients; N.S. = not significant.

**Table 4:** Values of the circulating amino acids concentrations (means ± SD) in Young non-DS Patients (spanning 30 to 60 years of age); Aged non-DS Patients (AnonDSP, spanning 75 to 90 years of age); Young DS Patients (YDSP, spanning 20 to 40 years of age); and Aged DS Patients (ADSP, spanning 41 to 60 years of age). Significances of the comparisons between YnonDSP and AnonDSP, and YDSP and ADSP of the values of each compound are indicated.

Compound	Concentrations in Serum of YnonDSP (*n* = 55) μmol/L Serum	Concentrations in Serum of AnonDSP (*n* = 47) μmol/L Serum	Significantly Different from YnonDSP *q*-Values	Concentrations in Serum of YDSP (*n* = 29) μmol/L Serum	Concentrations in Serum of ADSP (*n* = 27) μmol/l serum	Significantly Different from YDSP *q*-Values
ASP	52.65 ± 17.43	67.92 ± 17.47	<0.001	44.06 ± 10.47	51.91 ± 8.76	<0.005
GLU	71.92 ± 21.95	61.30 ± 13.14	<0.001	53.42 ± 14.55	38.20 ± 12.61	<0.001
ASN	66.21 ± 12.93	60.66 ± 8.59	<0.02	55.94 ± 13.19	53.54 ± 11.82	N.S.
SER	95.37 ± 22.16	90.29 ± 18.81	N.S.	80.81 ± 19.90	76.64± 13.65	N.S.
GLN	369.80 ± 77.15	358.85 ± 56.05	N.S.	458.10 ± 85.40	477.70 ± 70.40	N.S.
HIS	73.14 ± 19.21	67.51 ± 16.94	N.S.	65.61 ± 13.26	58.35 ± 14.11	N.S.
GLY	225.76 ± 79.84	212.83 ± 74.99	N.S.	202.71 ± 40.90	223.50± 70.19	N.S.
THR	129.48 ± 28.37	132.76 ± 34.48	N.S.	96.03 ± 28.21	95.29 ± 22.15	N.S.
CITR	26.50 ± 8.52	34.57 ± 8.05	<0.0001	31.31 ± 10.87	36.06 ± 8.89	<0.025
ARG	32.34 ± 9.15	33.80 ± 11.37	N.S.	42.81 ± 12.90	42.14 ± 11.20	N.S.
ALA	383.78 ± 102.42	358.14 ± 89.11	N.S.	366.34 ± 122.14	399.67 ± 115.63	N.S.
TAU	100.99 ± 22.60	87.88 ± 20.38	<0.0001	56.93 ± 20.20	56.04 ± 18.06	N.S.
TYR	77.11 ± 19.16	71.00 ± 15.69	N.S.	58.85 ± 11.87	52.96 ± 11.88	N.S.
VAL	274.21 ± 62.28	250.24 ± 47.47	N.S.	255.29 ± 54.88	239.58 ± 35.19	N.S.
MET	26.27 ± 5.35	24.60 ± 7.61	N.S.	21.62 ± 5.13	18.39 ± 3.24	<0.02.
TRP	64.42 ± 13.00	41.86 ± 11.52	<0.0001	52.04 ± 10.70	40.79 ± 13.48	<0.005
PHE	65.26 ± 13.00	57.95 ± 8.66	<0.0001	57.50 ± 11.49	52.99 ± 8.16	N.S.
ILE	73.18 ± 18.37	65.48 ± 22.56	N.S.	65.79 ± 15.26	59.31 ± 11.21	N.S.
LEU	139.22 ± 32.14	123.19 ± 37.32	<0.002	121.02 ± 27.11	110.04 ± 19.64	N.S.
ORN	151.40 ± 43.03	161.30 ± 44.04	N.S.	104.29 ± 31.20	88.14 ± 21.92	N.S.
LYS	203.09 ± 42.86	206.97 ± 53.70	N.S.	216.54 ± 49.25	199.33 ± 38.64	N.S.

ASP = aspartate, GLU = glutamate, ASN = asparagine, SER = serine, GLN = glutamine, HIS = histidine, GLY = glycine, THR = threonine, CIT = citrulline, ARG = arginine, ALA = alanine, TAU = taurine, TYR = tyrosine, VAL = valine, MET = methionine, TRP = tryptophan, PHE = phenylalanine, ILE = isoleucine, LEU = leucine, ORN = ornithine, LYS = lysine; YnonDSP = Young non-DS Patients; AnonDSP = Aged non-DS Patients; YDSP = Young DS Patients; ADSP = Aged DS Patients; N.S. = not significant.

## Data Availability

The data presented in this study are available on request from the corresponding author. The data are not publicly available due to restrictions of privacy.
